# Therapeutic use of a cationic antimicrobial peptide from the spider *Acanthoscurria gomesiana *in the control of experimental candidiasis

**DOI:** 10.1186/1471-2180-12-28

**Published:** 2012-03-06

**Authors:** Diego C Rossi, Julian E Muñoz, Danielle D Carvalho, Rodrigo Belmonte, Bluma Faintuch, Primavera Borelli, Antonio Miranda, Carlos P Taborda, Sirlei Daffre

**Affiliations:** 1 Department of Parasitology, Institute of Biomedical Sciences, University of São Paulo, Av. Prof Lineu Prestes, 1374, 05508-900 São Paulo, SP, Brazil; 2 Department of Microbiology, Institute of Biomedical Sciences and Laboratory of Medical Mycology IMT/SP - LIM53, University of São Paulo, São Paulo, SP, Brazil; 3 Department of Special Analysis, SD&W Modelagem e Soluções Estratégicas Ltda., São Paulo, SP, Brazil; 4 Radiopharmacy Center, Institute of Energetic and Nuclear Research, São Paulo, Brazil; 5 Department of Clinical and Toxicological Analyses, Faculty of Pharmaceutical Sciences, São Paulo University, São Paulo, SP, Brazil; 6 Departament of Biophysics, University Federal of São Paulo, São Paulo, SP, Brazil

## Abstract

**Background:**

Antimicrobial peptides are present in animals, plants and microorganisms and play a fundamental role in the innate immune response. Gomesin is a cationic antimicrobial peptide purified from haemocytes of the spider *Acanthoscurria gomesiana*. It has a broad-spectrum of activity against bacteria, fungi, protozoa and tumour cells. *Candida albicans* is a commensal yeast that is part of the human microbiota. However, in immunocompromised patients, this fungus may cause skin, mucosal or systemic infections. The typical treatment for this mycosis comprises three major categories of antifungal drugs: polyenes, azoles and echinocandins; however cases of resistance to these drugs are frequently reported. With the emergence of microorganisms that are resistant to conventional antibiotics, the development of alternative treatments for candidiasis is important. In this study, we evaluate the efficacy of gomesin treatment on disseminated and vaginal candidiasis as well as its toxicity and biodistribution.

**Results:**

Treatment with gomesin effectively reduced *Candida albicans *in the kidneys, spleen, liver and vagina of infected mice. The biodistribution of gomesin labelled with technetium-99 m showed that the peptide is captured in the kidneys, spleen and liver. Enhanced production of TNF-α, IFN-γ and IL-6 was detected in infected mice treated with gomesin, suggesting an immunomodulatory activity. Moreover, immunosuppressed and *C. albicans*-infected mice showed an increase in survival after treatment with gomesin and fluconazole. Systemic administration of gomesin was also not toxic to the mic

**Conclusions:**

Gomesin proved to be effective against experimental *Candida albicans* infection. It can be used as an alternative therapy for candidiasis, either alone or in combination with fluconazole. Gomesin's mechanism is not fully understood, but we hypothesise that the peptide acts through the permeabilisation of the yeast membrane leading to death and/or releasing the yeast antigens that trigger the host immune response against infection. Therefore, data presented in this study reinforces the potential of gomesin as a therapeutic antifungal agent in both humans and animals.

## Background

Antimicrobial peptides (AMPs) are components of the innate immune system of vertebrates and invertebrates, having a broad-spectrum activity against bacteria, fungi, viruses and protozoa [1]. In general, AMPs are small molecules with 1 to 10 kDa of molecular mass and exhibit a high content of basic amino acids, which results in an overall positive net charge. AMPs also usually have an amphipathic structure. Thus, while the positive charges of basic amino acids facilitate interaction with the negative charges of the phospholipids of biological membranes, the hydrophobic amino acids facilitate the insertion of AMPs into the membrane, which will eventually lead to lysis of the microorganisms. Some AMPs can act on internal targets, such as the inhibition of nucleic acid and/or protein synthesis [1,2]. Alternatively, some AMPs selectively boost the host immune response through the regulation of the production of proinflammatory cytokines and chemokines and by promoting the chemotaxis of T cells, monocytes, neutrophils and eosinophils. They can also effect dendritic cell differentiation and stimulate angiogenesis [3].

Gomesin is a cationic AMP isolated from haemocytes of the tarantula spider *Acanthoscurria gomesiana*[4]. This peptide contains 18 amino acids and two disulphide bridges and adopts a β-hairpin structure [5]. The disulphide bridges provide stability in mammalian serum and resistance to proteolysis [6]. Gomesin exerts a strong microbicidal activity against Gram-positive and Gram-negative bacteria, filamentous fungi, yeast, parasites and tumour cells through a mechanism of pore formation or "detergent like" action [4,7-9].

Candidiasis is an infection caused by fungi from the genus *Candida* and can affect the skin, eyes, oral cavity, oesophagus, gastrointestinal tract, vagina and vascular system of humans. Most infections occur in patients who are immunocompromised or debilitated [10]. Vulvovaginal candidiasis is the most common form of mucosal disease, affecting up to 75% of women (review by [11]). In Brazil, candidiasis has become a public health problem. It is the 3^rd ^leading cause of death from systemic mycosis in AIDS-negative patients. Records indicate an increase in mortality from an annual average of 39 deaths between 1996 and 1998 to 54 between 2005 and 2006. Taking in account the deaths of AIDS patients with underlying cases of candidiasis, the disease is the 2^nd ^leading cause of death from systemic mycosis, with 1,780 deaths in Brazil from 1996 to 2006 [12]. Nosocomial candidiasis is also a public problem in Brazil [13]. In the USA, *Candida *species are the fourth leading cause of nosocomial bloodstream infections in several hospitals and the mortality from 1997 to 2003 was approximately 0.4 deaths per 100,000 population per year (review by [14,15]). The leading treatment of *Candida *infections is done with polyenes (amphotericin and liposomal amphotericin), azoles (fluconazole and voriconazole) and echinocandins (caspofungine) [16]. Regardless of which antifungal drug is used, there is frequent treatment failure [16]. In this paper, we show the potential therapeutic use of gomesin in an experimental infection of *C. albicans*.

## Results

### Evaluation of the antifungal activity of gomesin *in vitro*

The minimum inhibitory concentration (MIC) of gomesin in the isolate 78 and strain ATCC 90028 was 5.5 μM and 11 μM, respectively, while the MIC of Fluconazole in the isolate 78 and strain ATCC 90028 was 186 μM and > 1.5 mM, respectively. In addition, we observed growth inhibition of the isolate 78 with the combined treatment of 0.6 μM gomesin and 3.5 μM fluconazole. Growth inhibition of strain ATCC 90028 was observed with the combined concentration of 1.3 μM gomesin and 14.3 μM fluconazole (Table 1). Furthermore, the fractional inhibitory concentration index (FICI) of the combination of gomesin and fluconazole was 0.11 in isolate 78 and 0.19 in strain ATCC 90028 (Table 1).

**Table 1:** Minimum inhibitory concentration (MIC) and fractional inhibitory concentration index (FICI) of gomesin and fluconazole

	MIC (μM)		FICI	
	
	*C. albicans *(78)	*C. albicans* (ATCC 90028)	*C. albicans *(78)	*C. albicans* (ATCC 90028)
Gomesin	5.5	11	-	-
Fluconazole	*	186	-	-
Gomesin + Fluconazole	0.6 + 3.5	1.3 + 14.3	0.11	0.19

* = not detected in up to 1.5 mM

### Evaluation of the antifungal activity of gomesin in mice with disseminated and vaginal candidiasis

**Figure 1:**
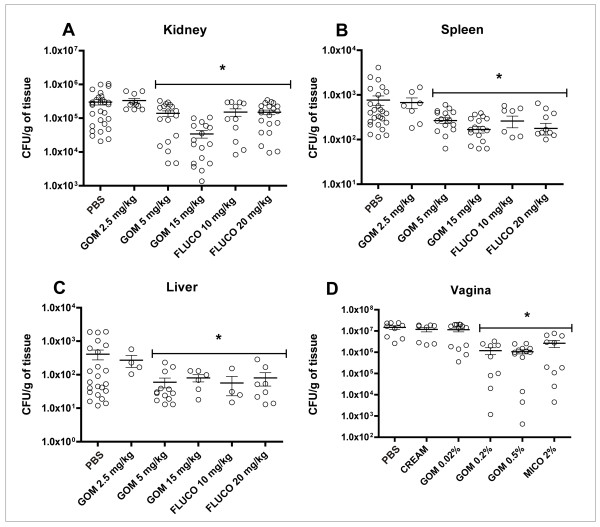
**Gomesin treatment of mice infected with *C. albicans***. Evaluation of the number of colony forming units (CFU) per gram of tissue of the kidneys (A), spleen (B), liver (C) and vagina (D). The disseminated candidiasis was performed by intravenous injection of 3 × 10^5 ^yeasts suspended in 100 μL of PBS and vaginal candidiasis was performed by inoculating 3 × 10^6 ^yeasts suspended in 20 μL of PBS. The treatment was done one, three and six days after infection with C. albicans (strain 78). Animals were treated with different doses of gomesin (GOM), fluconazole (FLUCO) and miconazole (MICO). As a control, infected animals received only PBS or cream (CREAM). * Indicates statistical significance (ANOVA with post-Tukey test, P < 0.05).

Treatment with 5 mg/kg and 15 mg/kg of gomesin in mice with disseminated candidiasis effectively reduced the fungal burden of the kidneys, spleen and liver when compared with the control group (PBS-treated mice) (Figure 1A-C). Treatment with 10 mg/kg and 20 mg/kg of fluconazole also effectively controlled the infection (Figure 1A-C). Moreover, treatment of vaginal candidiasis with 0.2% and 0.5% gomesin and 2% miconazole showed a significant decrease in colony forming units (CFUs) when compared with vehicle treatment (control group) (Figure 1D). The combination of gomesin and fluconazole or miconazole did not result in a synergistic effect.

### Cytokine levels in kidneys of gomesin-treated mice

**Figure 2:**
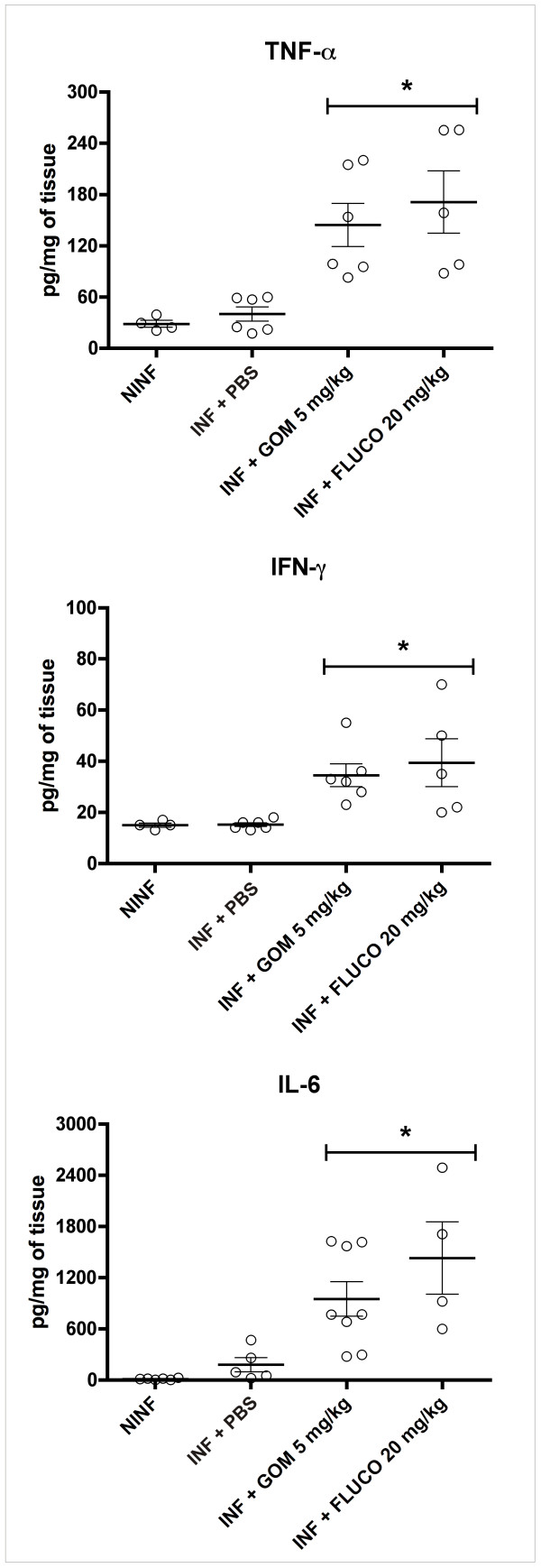
**Cytokine levels in kidneys**. Cytokine levels were evaluated in the kidneys of mice treated with gomesin (5 mg/kg) and fluconazole (20 mg/kg). Non-infected and untreated animals (NINF), as well as infected animals that received PBS, were used as controls. * Indicates statistical significance (*t*-test, P < 0.05) compared to the control INF.

Treatment with gomesin and fluconazole significantly increased the concentration of TNF-α, IFN-γ and IL-6 in the kidneys compared to controls that were not infected and not treated as well as controls that were infected and treated with PBS (Figure 2).

### Evaluation of the effect of antifungal drugs in immunosuppressed mice with disseminated candidiasis

**Figure 3:**
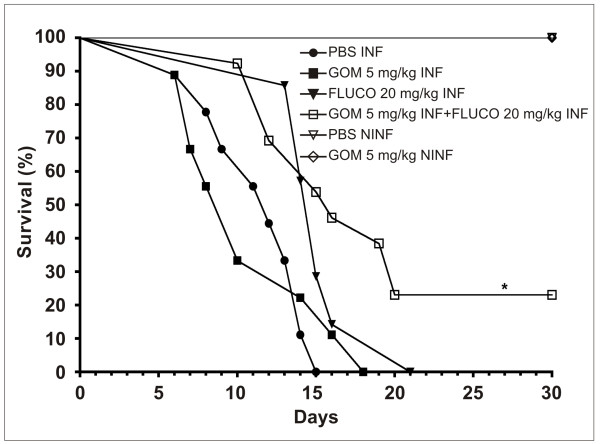
**Survival of immunosuppressed mice with disseminated candidiasis treated with antifungal drugs**. Animals were treated with 100 mg/kg of cyclophosphamide and infected with 10^3 ^yeasts of *C. albicans *(INF). The animals were treated with 5 mg/kg of gomesin (GOM), 20 mg/kg of fluconazole (FLUCO) or the combination of 5 mg/kg gomesin and 20 mg/kg of fluconazole. As controls, infected animals (NINF) received PBS and uninfected animals received PBS and gomesin 5 mg/kg. * Indicates statistical significance (Long-rank test, P < 0.05).

The group of infected animals that received PBS (control) reached 100% mortality on the fifteenth day after infection. No statistically significant difference was observed between the group treated with gomesin (5 mg/kg) and the group treated with fluconazole (20 mg/kg), although there was an increase in survival during the last treatment. Nonetheless, the combined treatment of 5 mg/kg of gomesin and 20 mg/kg of fluconazole produced a survival rate of 23% within 30 days after infection, which was statistically significant. The control groups that were not infected or those that received PBS or 5 mg/kg of gomesin remained alive until the end of the experiment (Figure 3).

### *In vivo* toxicity

Gomesin administration did not alter the number of leukocytes in the non-infected mice. However, when specific cell populations were analysed, the number of neutrophils and eosinophils were increased, whereas the number of lymphocytes was decreased. The administration of gomesin did not alter the haemoglobin levels. Nevertheless, treatment with gomesin resulted in an increase in the percentage of circulating reticulocytes. Moreover, the administration of gomesin showed no change in the levels of total bilirubin, direct and indirect, as well as creatinine and gamma-GT (Table 2).

**Table 2:** Evaluation of the toxicity of the gomesin treatment

	NINF*	NINF + GOM**
Leukocytes (mm^3^)	4637 ± 1114	4462 ± 1580
Neutrophils (mm^3^)	846 ± 288	1208 ± 388***
Eosinophils (mm^3^)	46 ± 46	135 ± 72***
Lymphocytes (mm^3^)	3744 ± 981	2660 ± 437***

Hemoglobin (g/dL)	13 ± 0.9	13 ± 0.5
Reticulocytes (%)	5.5 ± 0.7	9.3 ± 2.8***

Total Bilirubin (mg/dL)	0.48 ± 0.23	0.3 ± 0.1
Direct bilirubin (mg/dL)	0.35 ± 0.19	0.2 ± 0.1
Indirect bilirrubin (mg/dL)	0.13 ± 0.13	0.09 ± 0.009
Creatinine (mg/dL)	0.32 ± 0.09	0.34 ± 0.05
Gamma-GT (mg/dL)	< 1 U/L	< 1 U/L

* Non-infected mice

** Non-infected mice treated with gomesin (GOM)

*** *p *< 0.05

### Biodistribution of radiolabeled gomesin

**Figure 4:**
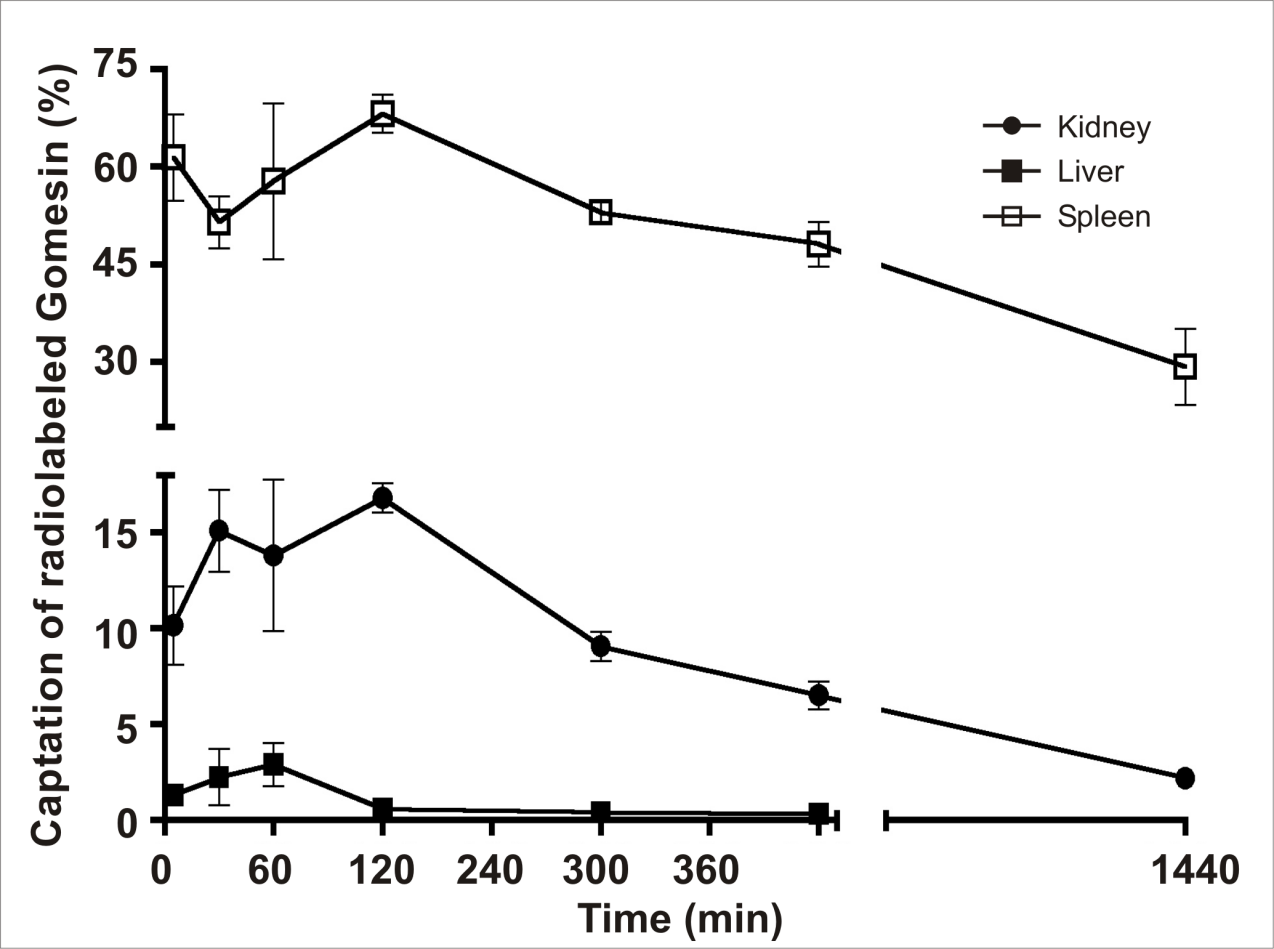
**Biodistribution of gomesin**. After administration of radiolabeled gomesin (99mTc-HYNIC-gomesin), the liver, kidneys and spleen were dissected at different time points to assess the biodistribution of the peptide.

The biodistribution of gomesin labelled with technetium-99 m was evaluated in the kidneys, spleen and liver (Figure 4). The liver had the highest percentage of radiolabeled peptide detected (60%), which persisted for up to 24 h post-injection, whereas the kidneys showed a radioactive peak at 120 min followed by a gradual decrease during the following hours. The spleen was the lowest of the organs tested (less than 5% detected) and was stable for only 60 min after administration of technetium-99 m-labelled gomesin, dropping to undetectable levels after 120 min.

## Discussion

Gomesin is an antimicrobial peptide isolated from haemocytes of the spider *Acanthoscurria gomesiana *and has a broad-spectrum of activity against bacteria, fungi, protozoa and tumour cells [4,7,9,17,18]. The antifungal activity of gomesin *in vitro* has previously been reported [4,7]. However, the antifungal activity against clinical isolates of *Candida albicans *resistant to antifungal drugs has not been studied. In this paper, we analysed the antifungal activity of gomesin *in vitro* and *in vivo* against a clinical strain of *C. albicans *(isolate 78), as well as its biodistribution and toxicity in mice.

Our data showed that *C. albicans *(isolate 78) is resistant to fluconazole up to 1.5 mM, but gomesin is effective against this strain at a lower concentration (MIC = 5.5 μM). This resistance to fluconazole is a common cause of treatment failure [19]. A synergism between gomesin and fluconazole against two isolates of *Candida albicans *(78 and ATCC 90028) was demonstrated using the FICI calculation method. The synergistic mechanism of gomesin and fluconazole is not completely understood, but studies with *Cryptococcus neoformans *suggested that gomesin, through membrane permeabilisation, promotes an increased entry of fluconazole into the fungal cytoplasm, which results in a better inhibition of the ergosterol synthesis. In this way, fluconazole is effective against *C. neoformans *at lower doses when applied in combination with gomesin [7]. A similar phenomenon was observed in murine melanoma cells (B16F10-Nex2) treated with gomesin and the monoclonal Mab A4M *in vitro*. The cytotoxicity of Mab A4M was only detected in the presence of gomesin, after permeabilisation of the cell membrane allowed the entry and action of the monoclonal antibody [9]. From these studies, we hypothesised that gomesin facilitates the entry of fluconazole in *Candida albicans *through membrane permeabilisation.

The literature on the use of antimicrobial peptides in the treatment of disseminated candidiasis is rather scarce. A study of the HLF peptide (1-11) originated from lactoferrin in immunosuppressed mice with disseminated candidiasis showed that a single dose of 0.4 ng/kg, 24 h after infection, was able to significantly reduce CFU in the kidneys [20]. ETD-151, an analogue of heliomicin also has been shown to be particularly effective against systemic candidiasis in comparison with amphotericin B and several azoles [21]. Likewise, treatment with gomesin proved to be effective against disseminated candidiasis. The peptide effectively reduced the fungal burden in the kidneys, which is the highest tropism organ for *Candida*. A similar effect was observed with fluconazole; however, this drug has some toxic effects and has selected resistance in *Candida albicans *[19]. Therefore, the use of gomesin as a therapeutic may be an alternative treatment for candidiasis because our results show that it is non-toxic in mice. Unlike *in vitro* treatment with gomesin and fluconazole, we have not detected any the synergistic effect of treatment with both drugs *in vivo*.

The treatment and prevention of recurrent vaginal candidiasis includes the use of imidazoles and triazoles as a first-line treatment, unless it is caused by a confirmed or suspected azole-resistant *Candida* strain. The efficacy of both oral and local therapy is similar, but, the local treatment presents several advantages, including a reduction of adverse effects; however, local treatment is contraindicated during pregnancy and breast feeding [22]. In recent years, there has been a focus on both understanding drug resistance to antifungal agents and optimising therapy of *Candida* infections [23]. There are no reports of topical treatment with antimicrobial peptides against vaginal candidiasis. In this paper, we are the first to describe an effective topical formulation of an antimicrobial peptide that is able to reduce CFUs count in an experimental vaginal candidiasis model. We found that 0.2% and 0.5% gomesin cream reduced the CFU on vaginas of the animals by 10 fold when compared to control animals. Minor changes in the treatment protocol with gomesin, either by increasing the frequency or changing the doses, may potentially produce better results. Treatment with 2% miconazole cream was also effective in controlling the CFUs of the vaginas of the animals. However, it was necessary to use a dose of miconazole that was at least four times higher than the dose of gomesin to produce a similar effect. No synergistic effect was observed after treatment with a combination of gomesin and miconazole.

In addition to the direct action of AMPs on microorganisms, either through membrane permeabilisation or internal target interference [2], it has been reported that some AMPs may possess an immunomodulatory function [3]. In order to verify if gomesin has such activity, the concentrations of IFN-γ, TNF-α and IL-6 were evaluated in the kidneys of mice that had been infected with *C. albicans *and treated with this peptide. These cytokines, especially IL-6, activate neutrophils, which play an essential role in the defence mechanism against *Candida*[24]. We observed that treatment with 5 mg/kg gomesin significantly increased the concentration of the three cytokines analysed. A similar effect was also observed with fluconazole treatment. The increase of cytokine levels in the kidneys might help to control candidiasis through the activation of the host immune system. This action appears to be similar to that observed with another AMP, murine β defensin-2, which acts via TLR4 and leads to the production of various cytokines, such as IL-12 and IL-6, as well as chemokines [25]. However, we cannot dismiss the hypothesis that the direct action of gomesin can trigger the release of pathogen-associated molecular patterns, or *PAMP*s, which would exacerbate the immune response of animals. This has been previously reported for the antimicrobial peptide human β defensin-2 [26]. The use of antimicrobial peptides as immunomodulatory agents for therapeutic application is an effervescent field in progress [27].

After verifying that the gomesin treatment was effective against disseminated candidiasis in healthy mice, we decided to evaluate the activity of gomesin in immunosuppressed animals, as candidiasis is typically observed in immunocompromised hosts [10]. Treatment with gomesin (5 mg/kg) showed no significant increase in survival compared to control animals. This suggests that the direct action of gomesin was not sufficient to control the infection and that immunomodulatory action is required to suppress the candidiasis. Treatment with fluconazole (20 mg/kg) also did not result in a significant increase in the survival of treated animals as compared to control animals. However, the combined treatment of 5 mg/kg gomesin and 20 mg/kg of fluconazole resulted in 23% survival of mice 30 days after infection. This could be due to gomesin facilitating the entry of fluconazole into the yeast, thus leading to the survival of animals. Another hypothesis is that treatment with fluconazole, being fungistatic, would allow time for gomesin to act.

To evaluate whether gomesin could be used as a therapeutic treatment for *C. albicans* infection, we performed blood analyses to determine the toxicity of gomesin in mice. No difference in the total number of leukocytes was observed in animals treated with gomesin. However, the number of eosinophils in mice not infected with *Candida albicans* but treated with gomesin was higher than the control group. The eosinophilia caused by gomesin may be due to the induction of an allergic response. Further experiments are needed in order to evaluate this effect. We have also noticed that gomesin treatment leads to a higher number of neutrophils. This effect might be a consequence of the induction of the pro-inflammatory response by gomesin, which would stimulate the bone marrow to recruit neutrophils. However it is not currently known if these cells are being recruited to the site of infection.

In addition, gomesin did not change the haemoglobin levels, which suggests that this peptide was not toxic to erythrocytes. However, the quantity of reticulocytes is greater in treated animals, suggesting that the peptide provokes an erythropoiesis compared to control animals (non-gomesin treated). Perhaps treatment with gomesin causes hypoxia in animals, thus increasing erythropoietin [28]. Furthermore, gomesin was not nephrotoxic or hepatotoxic, as the bilirubin, creatinine, and Gamma GT levels from treated animals are similar to the control group. Therefore, gomesin seems to be non-toxic to mice.

In addition to the evaluation of toxicity, the biodistribution of gomesin was performed to understand its pharmacokinetics and therefore its therapeutic potential. The biodistribution data revealed that the peptide mainly accumulates in the liver, although it also accumulates in the kidneys and spleen, within the first several minutes after administration. This suggests a rapid clearance from the circulation. The presence of gomesin in the sites of infection might explain the reduction of *Candida albicans *observed in our experiments. However, other studies are needed to monitor the excretion of the peptide.

## Conclusions

Gomesin was effective against *Candida albicans* infection *in vitro* and *in vivo*. Gomesin can be used as an alternative treatment for candidiasis, either alone or in combination with fluconazole. Although the mechanism of action of gomesin is not fully understood, it has been suggested that it directly acts on the fungal membrane and/or stimulates the immune response against yeast infection. Data presented in this study reinforces the potential of gomesin as a therapeutic antifungal agent in both humans and animals.

## Methods

### Antimicrobial compounds

The chemically synthesised gomesin was obtained from GENEPEP (France) with 97% purity analysed by liquid chromatography - mass spectrometry. Fluconazole was obtained from Pfizer (Pfizer Inc., New York) and miconazole from Janssen Pharmaceutica (Janssen-Pharmaceutica, Beerse). Gomesin and fluconazole were dissolved in PBS for the *in vivo* assays and water for *in vitro* tests. Miconazole was dissolved in PBS with 20% dimethyl sulfoxide (DMSO) for incorporation into the vaginal cream.

### *Candida albicans *strains 

Two strains of *Candida albicans *were used: isolate 78 [29] and the isolate ATCC 90028. Periodically, isolate 78 was inoculated into mice in order to maintain its virulence.

### *In vitro* studies

The antifungal activity of antimicrobial compounds was evaluated by using the protocol M-27A2, according to the Clinical and Laboratory Standards Institute (CLSI) [30]. Briefly, 80 μl of RPMI 1640 with 1.6 M MOPS pH 7 containing 10^4 ^yeast/mL of *C. albicans* in logarithmic growth phase, were added to the wells of a polypropylene 96-well plate containing 20 μl of serial two-fold dilution of gomesin (starting at 44 μM), fluconazole (starting at 1,488 μM) or the combination of gomesin (starting at 11 μM) and fluconazole (starting at 115 μM). After 48 h of incubation at 37°C fungal growth was evaluated by determining the absorbance at 595 nm. The lowest concentration that inhibited 100% growth was considered the minimum inhibitory concentration (MIC). The fractional inhibitory concentration index (FICI) was determined following the methodology described previously [31].

### Animals

BALB/c mice (6- to 8-week-old males or females) were bred at the Animal Facility at the Institute of Biomedical Science of University of São Paulo, Department of Immunology under specific pathogen-free conditions. Food and water were given ad libitum. All animals were handled in accordance with good animal practice as defined by the relevant national animal welfare bodies and all *in vivo* testing was approved by the Institutional Animal Care and Use Committee of the University of São Paulo, reference number: 87/42. For immunosuppression of animals, doses of 100 mg/kg cyclophosphamide were administered intraperitoneally 4 days and 1 day before infection with *C. albicans*, the third day after infection and, from this point on, every 4 days until the end of treatment [32]. The animals were kept in cages lined with wood shavings and closed with autoclaved filter, and served autoclaved food and water in order to maintain a sterile environment. Cages were exchanged twice a week in laminar flow hoods. The animals were considered anergic when the number of leukocytes was found to be less than 100 cells/mm^3 ^[33]. The vaginal candidiasis model was developed by inducing the pseudo oestrus phase by the subcutaneous administration of 0.5 mg of 17 beta-estradiol valerate (Sigma Chemicals, St Louis), dissolved in sesame oil (Sigma Chemicals, St Louis) 3 days before the vagina's infection [34]. Swiss mice were provided by the Animal Facility of IPEN-CNEN for the biodistribution studies.

### Infections

One colony of *C. albicans *(isolate 78) was selected from the plate dishes and incubated in brain heart infusion (Oxoid, England) at 37°C for 24 h with 200 rpm agitation. The sediment obtained by centrifugation at 1500 g for 5 min was washed three times in PBS and resuspended in 5 mL of PBS. The number of yeast per mL of this suspension was determined with a Neubauer chamber.

The disseminated candidiasis was induced by intravenous injection of 3 × 10^5^/100 μL of PBS and the immunosuppressed model was induced by intravenous injection of 10^3 ^yeasts suspended in 100 μL of PBS. Vaginal candidiasis was induced by inoculating 3 × 10^6 ^yeasts suspended in 20 μL of PBS.

### *In vivo* treatments

Mice with disseminated candidiasis were treated with gomesin and fluconazole. The drugs were administered intraperitoneally in a final volume of 500 μl at the following concentrations: gomesin (2.5 mg/kg, 5 mg/kg and 15 mg/kg), fluconazole (10 mg/kg and 20 mg/kg) and a combination of both (2.5 mg/kg to 5 mg/kg gomesin and 10 mg/kg to 20 mg/kg fluconazole). For mice with vaginal candidiasis, gomesin (0.02%, 0.2% and 0.5%), miconazole (2%) and a combination of both (0.2% gomesin and 2% miconazole) were incorporated into a vaginal cream (10% Wax self-nonionic emulsifier, 2% mineral oil, 5% propylene glycol and 84% distilled water, pH 4.5) for topical application. For all treatments, drugs were administered 1, 3 and 6 days after infection with *C. albicans*. An equivalent volume of PBS or cream was administered to the control animals. To evaluate the fungal burden, the kidneys, spleen, liver and vagina of the mice were dissected aseptically on the seventh day after infection, weighed and homogenised in 1 mL of PBS. Aliquots of the homogenate (100 μl) were inoculated on brain and heart infusion (Oxoid, England) containing 2% agar. After incubation for 18 h at 37°C, the number of CFUs was determined. The effectiveness of treatment was determined by comparing the number of CFUs per gram of tissue of treated animals with the number of CFUs per gram of tissue of control animals (untreated). For the survival curves, the animals were monitored daily for 30 days.

### Measurement of cytokines

The kidneys of animals infected with *C. albicans *and treated with gomesin or fluconazole were dissected, washed in PBS and homogenised with an electric tissue homogeniser in 1 mL of PBS containing a cocktail of protease inhibitors (Sigma Chemicals, St Louis). This cocktail of protease inhibitors was composed of 1 μg/ml of pepstatin A (aspartyl protease inhibitor), 4 mM benzamidine (seine protease inhibitor), 1 mM ethylenediaminetetraacetic acid acetic (metallo-protease inhibitor) and 1 mM N-Ethylmaleimide (cysteine protease inhibitor). Non-infected animals or animals infected and not treated were used as controls. The concentrations of IFN-γ, TNF-α and IL-6 were evaluated on a flow cytometer (BD FACSCaliburTM, San José) using the kit Cytometric Bead Array and Mouse Inflammation™ (BD, San José) and the methodology described by the manufacturer.

### Blood analysis

Blood was collected by puncturing the brachial plexus of anesthetised mice using EDTA (1%) as an anticoagulant after 7 days of gomesin administration (15 mg/kg). Reticulocytes cells and leukocytes were counted by standard methods. The haemoglobin concentration was determined using the modified Drabkin method. Blood samples were prepared on microscopic glasses, dried and stained with May-Grünwald reagents for morphological examination of the blood. The number of reticulocytes was determined in blood smears stained with Supra Vital New Methylene Blue. We also determined the levels of bilirubin, creatinine and gamma GT biochemically using the Sims-Horm, Enzyme and Alkaline picrate methods, respectively.

### Evaluation of the biodistribution of radiolabelled gomesin with technetium-99 m in mice

HYNIC-gomesin was manually synthesised by solid phase methodology as described previously, except that pyroglutamic acid was substituted for 6-hydrazino nicotinamide (HYNIC) [6]. The HYNIC-gomesin conjugate was labelled with the radioisotope technetium-99 m obtained from an alumina-based 99Mo/99mTc generator, supplied by the Radiopharmacy Centre of the Institute of Energetic and Nuclear Research (IPEN/CNEN). Briefly, 20 mg of tricine and 5 mg of ethylenediamine N,N'-diacetic acid (EDDA) were dissolved in 0.5 ml of 0.1 M PBS, previously nitrogenated. Ten micrograms of HYNIC-gomesin, 5 μl of 8.9 mM SnCl_2_·2H_2_O solution in 0.1 N HCl (nitrogen-purged) and 500 μl of Na^99m^TcO_4 _was added to the vial. The reaction was conducted by heating the solution at 100°C for 20 min in a water bath and then allowing it to cool to room temperature. The pH of the reaction mixture was 7 [35]. The product ^99m^Tc-HYNIC-gomesin (0.1 mL), with an approximate activity of 74 MBq (2 mCi), was administered to the tail vein of the mice. The animals were sacrificed in a CO_2 _chamber at 5, 30, 60, 120, 240, 360, and 1,440 min after injection of the radiolabeled gomesin. Six animals were used for each time point. The kidneys, spleen and liver of each animal was dissected and transferred to tubes to measure radioactivity. The radioactivity count was performed in the Gamma Counter Shaft type NaI, using the same standard dose in injected animals minus the radioactivity retained in the site injection (tail). The uptake of radiolabeled gomesin by each organ was calculated using the following equation: DI% = (CPM organ/standard CPM) × 100), where%DI = percentage of the injected dose and CPM = count per min [35].

### Statistical analysis

ANOVA, with the post-Tukey test, was used to evaluate the statistical significance of results obtained in all experiments except the blood and survival analysis, where the Students *t*-test and Log-rank test were used, respectively. The differences between the results obtained with treatment compared to the controls were considered statistically significant when the p value was less than 0.05.

## Authors' contributions

DR carry out *in vitro*, *in vivo* studies and measurement of cytokines, participated in biodistribution experiments, blood analysis, experimental design and helped to write the manuscript. JEM participated in the *in vivo*, *in vitro* studies and measurement of cytokines. RB participated in *in vivo* and *in vitro* studies. AM synthetised the HYNIC-gomesin. BF was responsible for the biodistribution experiments. PB carries out the blood analysis. DDC was in charge of the statistical analysis. CPT and SD conceived of the study and manuscript preparation. All authors read and approved the final manuscript.
